# S45. MENTALIZATION BASED TREATMENT FOR NON-AFFECTIVE PSYCHOTIC DISORDER

**DOI:** 10.1093/schbul/sby018.832

**Published:** 2018-04-01

**Authors:** Jonas Weijers, Corine ten Kate, Elisabeth Eurelings-Bontekoe, Wolfgang Viechtbauer, Jean-Paul Selten

**Affiliations:** 1 GGZ Leiden; 2 GGZ Rivierduinen; 3 Leiden University; 4 Maastricht University; 5 Maastricht University, Rivierduinen Mental Health Care Institute

## Abstract

**Background:**

Deficits in mentalizing – i.e. the ability to understand one’s own and another’s behavior in terms of mental states such as beliefs, feelings and intentions – have been widely observed in patients with non-affective psychotic disorder (NAPD). In turn, robust evidence has shown these impairments to be related to social dysfunction and negative symptoms. However, few treatments have been developed to effectively treat impaired mentalizing, in spite of its increased recognition as an important treatment target. Mentalization based treatment (MBT) is a psychodynamic therapy rooted in attachment theory, originally developed and empirically found to be effective in treating borderline personality disorder. MBT for psychotic disorder aims to improve social functioning in NAPD patients by targeting impaired mentalizing.

**Methods:**

The study is a multicenter, rater-blinded, randomized controlled trial. Ninety patients, who were diagnosed with NAPD by a psychiatrist and whose diagnosis was confirmed by researchers with the CASH, were recruited from community treatment teams in the Netherlands. They were randomly allocated to either treatment as usual plus MBT or to treatment as usual only. MBT consisted of 18 months of group therapy (one hour weekly) and individual therapy (30 minutes per two weeks). Patients had a mean age of 31.48 years (SD = 8.87) and a mean duration since onset of psychosis of 5.53 years (SD = 3.65). The primary outcome variable was social functioning (measured with the Social Functioning Scale). Other outcome variables were positive, negative, depressive, and anxious symptoms, as well as insight (PANSS), quality of life (MANSA), substance abuse, social stress reactivity (Experience Sampling Method), and mentalizing capacity (Social Cognition and Object-Relations System; Hinting Task).

**Results:**

This will be the first presentation of our trial results.

**Discussion:**

The clinical implications of the results and limitations of the trial will be discussed. Theoretical considerations suggest that mentalization-based treatment could be an effective treatment for social dysfunction and impaired mentalizing in NAPD. If Mentalization-based treatment for psychotic disorders proves to be effective at improving social functioning and mentalizing, it may provide a valuable addition to treatment as usual.

